# Effectiveness of Cognitive Orientation to Occupational Performance intervention in improving motor skills of children with developmental coordination disorder: A randomized waitlist-control trial

**DOI:** 10.1177/02692155221086188

**Published:** 2022-04-24

**Authors:** Sara Izadi-Najafabadi, Cassandra Gunton, Zara Dureno, Jill G Zwicker

**Affiliations:** 1 Graduate Programs in Rehabilitation Sciences, 8166 University of British Columbia, Vancouver, Canada; 2 512469 BC Children’s Hospital Research Institute, Vancouver, Canada; 3 Department of Occupational Science & Occupational Therapy, University of British Columbia, Vancouver, Canada; 4 Department of Pediatrics, 8166 University of British Columbia, Vancouver, Canada; 5 Sunny Hill Health Centre at BC Children’s Hospital, Vancouver, Canada; 6 CanChild Centre for Childhood Disability Research, Hamilton, Canada

**Keywords:** Randomized controlled trial, paediatric rehabilitation, occupational therapy, motor skills disorder, developmental coordination disorder

## Abstract

**Objectives:**

To determine if Cognitive Orientation to Occupational Performance was effective in improving performance and transfer of motor learning in children with developmental coordination disorder (with/without attention deficit hyperactivity disorder); and whether outcomes were maintained three months post-intervention.

**Design:**

Randomized waitlist-control trial (ClinicalTrials.gov ID: NCT02597751)

**Setting:**

BC Children's Hospital, Vancouver, Canada

**Subjects:**

Thirty-seven children with developmental coordination disorder and 41 children with co-occurring attention deficit hyperactivity disorder (all 8–12 years), randomized to treatment or waitlist groups.

**Interventions:**

One-hour of intervention once weekly for 10 weeks.

**Main Measures:**

(1) Canadian Occupational Performance Measure to measure self-perceived performance of motor goals (10-point scale); (2) Performance Quality Rating Scale to measure therapist-observed movement quality (10-point scale); and (3) Bruininks-Oseretsky Test of Motor Proficiency – 2nd ed. to measure overall motor skill ability/transfer of motor learning (percentile).

**Results:**

Both groups showed significant improvement (*p* < 0.001) in motor performance (developmental coordination disorder: pre: 2.7 ± 2.2, post: 7.0 ± 1.0; developmental coordination disorder with attention deficit hyperactivity disorder: pre: 2.3 ± 1.7, post: 7.0 ± 1.5) and movement quality (developmental coordination disorder: pre: 3.0 ± 1.5, post: 6.3 ± 1.7; developmental coordination disorder with attention deficit hyperactivity disorder: pre: 3.0 ± 1.9, post: 5.7 ± 2.3). Three months after treatment, children maintained their gains, but only children with developmental coordination disorder showed transfer of learning to overall motor skills (pre:12 ± 15, post:12 ± 12, follow-up:14 ± 20, *p* < 0.001).

**Conclusion:**

Intervention was similarly effective for children with developmental coordination disorder with/without attention deficit hyperactivity disorder in achieving and maintaining functional motor goals, but only children with developmental coordination disorder showed transfer of learning to other motor skills.

## Introduction

Developmental coordination disorder is characterized by slow, variable, and less accurate motor performance and difficulty with motor learning,^1^ which in turn, significantly interferes with activities of daily living, academic achievement, and leisure activities.^2^ Without intervention, up to 75% of children with developmental coordination disorder continue to have difficulties as adults,^3^ which emphasizes the necessity of accessing early, effective, and efficient intervention services for these children. Over 50% of children with developmental coordination disorder have co-occurring attention deficit hyperactivity disorder.^4^ When these disorders occur together, the motor difficulties in the child's life are exacerbated.^5^

Children with co-occurring developmental coordination disorder and attention deficit hyperactivity disorder have a significantly higher risk of psychological distress,^6^ antisocial behaviour,^5^ and reduced participation in activities that require motor coordination and attention, resulting in overall lower quality of life than children with a single diagnosis.^7^ Adolescents with co-occurring conditions also experience high levels of peer victimization^8^ and are the least popular in the class.^9^ Since ADHD is mainly characterized by a patterns of hyperactivity and inattention inconsistent with child's developmental level, they often get treated only for their attention difficulties and their motor deficits get ignored.^10,11^ Only half of children with ADHD receive motor-based treatment.^10,11^

The Cognitive Orientation to daily Occupational Performance approach is an individualized, task-specific, cognitive-based, problem-solving approach for individuals experiencing difficulties performing the skills they want or need to do.^12^ While a few studies have been conducted to evaluate the effectiveness of Cognitive Orientation to daily Occupational Performance in skill acquisition,^13,14^ a high quality and large RCT is needed to further confirm its effectiveness and its longer-term effect for children with developmental coordination disorder with or without co-occurring conditions. Moreover, how task transfer happens in children with developmental coordination disorder is not yet clear and needs further investigation. Thus, in this study, we aimed to further understand if Cognitive Orientation to daily Occupational Performance can be transferred to foundational skills and general motor skill ability.

Thus, the aims of this study were to determine: (1) if Cognitive Orientation to daily Occupational Performance is effective in improving the self-rated performance and satisfaction of functional motor goals and motor quality; (2) if Cognitive Orientation to daily Occupational Performance can be transferred to or improve overall motor skill ability; and (3) whether outcomes are maintained three months post-intervention in children with developmental coordination disorder (with and without co-occurring attention deficit hyperactivity disorder). Based on previous evidence,^13,14^ we expected to see improved performance and satisfaction scores and higher movement quality after CO-OP intervention, and that these gains would be maintained at follow-up. However, based on principles of neuroplasticity^15^ (e.g. “gain what you train”), we hypothesized that there would be no transfer of learning to other motor skills.

## Methods

This study reports behavioural results of a randomized waitlist control trial (RCT) (ClinicalTrials.gov ID: NCT02597751) investigating brain changes^16,17^ associated with Cognitive Orientation to daily Occupational Performance intervention. From September 2014 to July 2019, 115 children (35 typically-developing children, 37 children with developmental coordination disorder, and 43 children with developmental coordination disorder with co-occurring attention deficit hyperactivity disorder) were recruited in Greater Vancouver area from the Developmental Coordination Disorder Clinic at Sunny Hill Health Centre, BC Children's Hospital Attention Deficit Hyperactivity Disorder Clinic, caseloads of occupational and/or physical therapists from Sunny Hill and the Vancouver Regional Pediatric Team, and from advertisements in the community. Developmental coordination disorder diagnosis was confirmed as per the Diagnostic and Statistical Manual – 5th ed.^2^ diagnostic criteria and other international guidelines.^18^

Participants were enrolled in the study after parental consent and child assent as per ethics approval (H14-00397) from the University of British Columbia/BC Children's and Women's Research Ethics Board. For the purpose of this study, we did not include any typically developing children.

**Figure 1.**
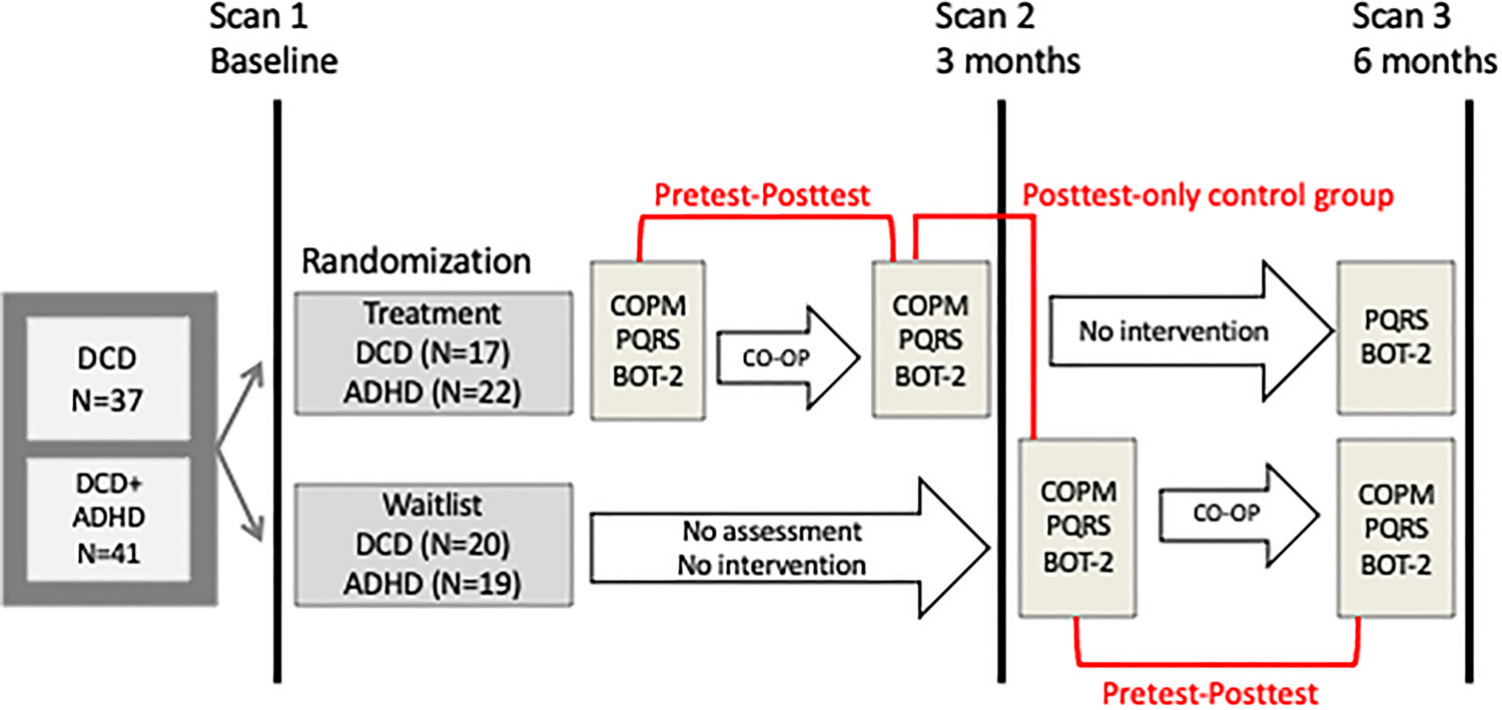
Schematic of study design. [Adapted by permission from: SpringerLink Nature Switzerland, Current Developmental Disorder Reports, Training-induced neuroplasticity in children with developmental coordination disorder by Izadi-Najafabadi *et al*., Copyright © 2020]. ADHD: attention deficit hyperactivity disorder; BOT-2: Bruininks-Oseretsky Test of Motor Proficiency-2^nd^ edition.; CO-OP: Cognitive Orientation to daily Occupational Performance; COPM: Canadian Occupational Performance Measure; DCD: developmental coordination disorder; PQRS: Performance Quality Rating Scale.

Participants were randomized into two groups – treatment and waitlist – using computer-generated sequential blocks of 4 to 6 done by a statistician. Opaque, sealed envelopes were used for allocation concealment until the first scan. Children in both groups were scanned upon enrollment, after three months, and after six months. Children in the treatment group received motor assessments and 10 weeks of Cognitive Orientation to daily Occupational Performance intervention in the first three months after enrollment, and then re-assessment three months post-intervention. Children in the waitlist group waited three months before completing the motor assessments and intervention. Figure 1 is a schematic of the study design^16^ and its associated behavioural analysis.

An independent occupational therapist, not involved in the intervention, used the Canadian Occupational Performance Measure,^19^ the Performance Quality Rating Scale,^20^ and the Bruininks-Oseretsky Test of Motor Proficiency – 2^nd^ ed^21^ (short form) to assess children's motor performance and satisfaction, motor quality, and general motor skills before and after the intervention. A two-point change in self-rated motor performance and satisfaction as assessed by Canadian Occupational Performance Measure is considered clinically significant.^22,23^ A change in score of 3 points using the generic rating system of the Performance Quality Rating Scale is also considered clinically significant.^20^ During the pre-intervention assessment session, children used the Pediatric Activity Card Sort (PACS)^24^ to select three personal motor goals to be addressed over the course of treatment. See Supplementary Table 1 for more information regarding child-chosen motor goals.

The Cognitive Orientation to daily Occupational Performance intervention was administered by 22 registered occupational therapists trained in the research protocol. Children were seen once weekly for one hour (∼20 min on each self-chosen motor goal) over 10 weeks as per published protocol^25^ at the Sunny Hill Health Centre for Children or BC Children's Hospital. Therapists kept detailed notes of strategies used for each goal, providing evidence of treatment fidelity. Throughout the intervention, the child was taught the global strategy of *Goal-Plan-Do-Check*. The child was encouraged to state what motor task they wanted to do (*Goal*), plan what strategies to use (*Plan*), try the strategies (*Do*), and evaluate how they worked *(Check*). Therapists used dynamic performance analysis to determine where the performance broke down during each task. They helped the child discover specific cognitive strategies (e.g. attention to the task, body positioning) to improve motor-based performance problems in their specific chosen activities.^13,26^ Since in-therapy sessions may not be sufficient for transfer of learning to other tasks,^27^ parents also received training to apply Cognitive Orientation to daily Occupational Performance strategies at home. They were required to attend the first treatment session so that therapists could instruct them on how to facilitate strategy use between treatment sessions. Parents' attendance in subsequent intervention sessions was encouraged but not mandated. Parents were provided with a logbook at the first session and were encouraged to practice child-chosen goals at home between treatment sessions and keep a record of the days and the amount of time they practiced at home.

To investigate the behavioural effect of intervention in children with developmental coordination disorder (with and without attention deficit hyperactivity disorder), we used pretest-posttest and posttest-only control group analyses. The pre- and post-intervention data used in this study were combined from children in both the waitlist and treatment groups. Statistical analyses were conducted using IBM SPSS Statistics version 23 (IBM, NY, USA). Since all outcome measures were ordinal – Canadian Occupational Performance Measure and Performance Quality Rating Scale are Likert scales and Bruininks-Oseretsky Test of Motor Proficiency – 2^nd^ ed. percentile scores are ranked variables – non-parametric statistical tests were used to answer our research questions. Wilcoxon Signed-Rank Test and the Mann-Whitney U test were used to compare the changes (post-intervention–pre-intervention score) in all outcome measures between the two groups. To determine if the treatment was effective three-months post-intervention, the non-parametric Friedman Test was used to compare pre-intervention, post-intervention, and follow-up assessments. To deal with randomization errors and make an unbiased conclusion, we used an intention-to-treat approach. More details about sample size calculation, participants inclusion/exclusion criteria, outcome measures, and statistical analysis can be found in supplementary material.

## Results

### Participants

**Figure 2.**
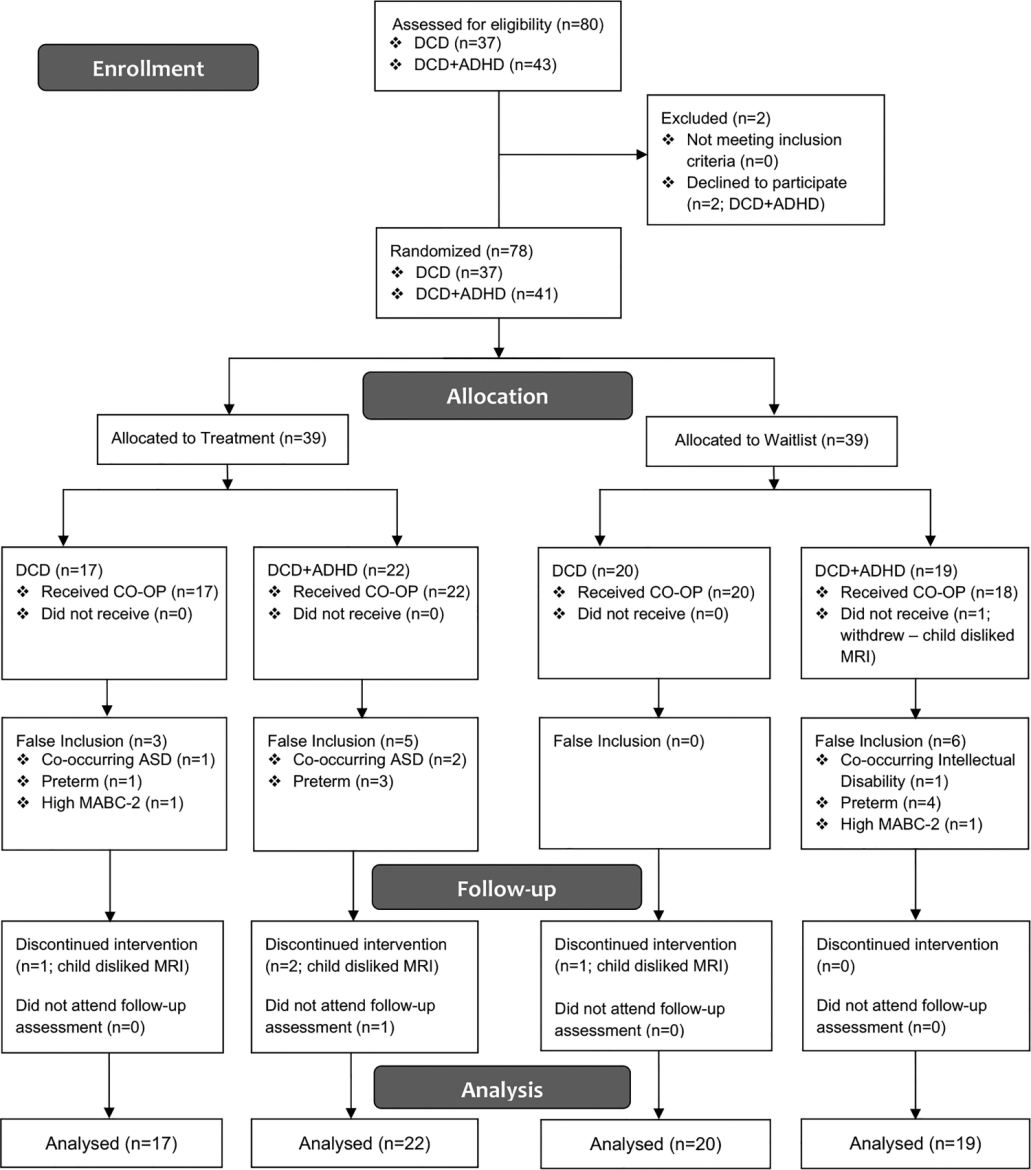
CONSORT flowchart of participants. ADHD: attention deficit hyperactivity disorder; ASD: autism spectrum disorder; CO-OP: Cognitive Orientation to daily Occupational Performance; DCD: developmental coordination disorder; MABC-2: Movement Assessment Battery for Children – 2^nd^ ed.

As shown in Figure 2, the CONsolidated Standards Of Reporting Trials (CONSORT) flowchart, from a total of 80 children recruited for this study, 37 children with developmental coordination disorder [25 male, 12 female; mean (SD) age: 9.7 (1.5) years] and 41 children with developmental coordination disorder with co-occurring attention deficit hyperactivity disorder [38 male, 3 female; mean (SD) age: 10.2 (1.4) years] participated in this study; two children with developmental coordination disorder with co-occurring attention deficit hyperactivity disorder declined to participate in the study due to claustrophobia (related to the MRI portion of the study) and disliking the MRI. Children in each group were randomized into treatment (17 developmental coordination disorder and 22 developmental coordination disorder with co-occurring attention deficit hyperactivity disorder) and waitlist groups (20 developmental coordination disorder and 19 developmental coordination disorder with co-occurring attention deficit hyperactivity disorder). None of these participants were excluded from the study or analysis due to intention-to-treat approach for data analysis. See Table 1 for participant characteristics including age, sex, clinical assessment scores as well as number of children taking attention deficit hyperactivity disorder-related medications (e.g. Biphentin, Concerta, Adderall, Vyvanse, and Fluxetine) in each group. Supplementary Table 2 shows demographic characteristics of all children with developmental coordination disorder (*n* = 78) in treatment group and waitlist group.

**Table 1.** Participant characteristics.

Variable	DCD (*n* = 37)		DCD + ADHD (*n* = 41)		*p-value*
	Treatment (*n* = 17)	Waitlist (*n* = 20)	Treatment (*n* = 22)	Waitlist (*n* = 19)	
Male Sex Assigned at Birth; N (%)	11 (65)^1,2^	14 (70)^3^	20 (91)^1^	18 (95)^2,3^	**0.04**
Age (years); Mean (SD)	10.1 (1.7)	9.4 (1.3)	10.0 (1.3)	10.5 (1.4)	0.11
DCDQ (total); Mean (SD)	27 (7.5)	32 (9.4)	29 (10.4)	32 (6.9)	0.24
MABC-2 (percentile); Median (IQR)	2 (8.3)	2 (4.5)	1 (2.3)	5 (8.5)	0.11
Conner's ADHD Index (t-score); Median (IQR)	90 (17)	88 (25)^4^	90 (3)	90 (0)^4^	**0.007**
ADHD-related Medications; N (%)	0 (0)^5,6^	4 (20)^7,8^	11 (50)^5,7^	11 (58)^6,8^	**<0.001**

ADHD: attention deficit hyperactivity disorder; DCD: developmental coordination disorder; DCDQ: Developmental Coordination Disorder Questionnaire; IQR: inter-quartile range; MABC-2: Movement Assessment Battery for Children – 2^nd^ edition; SD: standard deviation.

^1,2,3,4,5,6,7,8^ *p* < 0.05.

### Pretest-Posttest analysis

The results from the pre-post analysis demonstrated a statistically significant (*p* < 0.001) improvement in self-rated performance and satisfaction of motor goals on the Canadian Occupational Performance Measure and Performance Quality Rating Scale, but not the Bruininks-Oseretsky Test of Motor Proficiency – 2^nd^ ed. (Table 2). Results demonstrated a clinically significant (> 2-point) increase in self-perceived motor performance and satisfaction, respectively, for 83% to 86% of children with developmental coordination disorder as well as 90% to 92% of participants with developmental coordination disorder with co-occurring attention deficit hyperactivity disorder. Approximately 60% of children with developmental coordination disorder and 46% of children with developmental coordination disorder with co-occurring attention deficit hyperactivity disorder showed a clinically significant increase of > 3 points in Performance Quality Rating Scale scores.

**Table 2.** Outcomes before and after Cognitive Orientation to daily Occupational Performance intervention: pretest-posttest analysis.

Variable	DCD (*n* = 37)				DCD + ADHD (*n* = 41)			
	Pre-test Median (IQR)	Post-test Median (IQR)	Effect size	Median 95% CI	Pre-test Median (IQR)	Post-test Median (IQR)	Effect size	Median 95% CI
COPM Performance	2.7 (2.2)	7.0 (1.0)	0.62*	3.00–4.33	2.3 (1.7)	7.0 (1.5)	0.61*	3.83–4.67
COPM Satisfaction	3.0 (2.1)	8.0 (2.1)	0.61*	3.83–5.50	2.7 (2.7)	8.0 (2.0)	0.61*	3.97–5.33
PQRS	3.0 (1.5)	6.3 (1.7)	0.61*	2.83–3.67	3.0 (1.9)	5.7 (2.3)	0.60*	2.33–3.17
BOT-2 (percentile)	12 (15.0)	16 (20.0)	0.31	1.00–6.00	12 (18.8)	18 (23.5)	0.25	1.00–9.12

ADHD: attention deficit hyperactivity disorder; BOT-2: Bruininks-Oseretsky Test of Motor Proficiency – 2nd edition; CI: confidence interval; COPM: Canadian Occupational Performance Measure; DCD: developmental coordination disorder; IQR: inter-quartile range; PQRS: Performance Quality Rating Scale.

* Bonferroni-corrected *p *< 0.001.

There were no significant differences between children with developmental coordination disorder and children with developmental coordination disorder with co-occurring attention deficit hyperactivity disorder when comparing changes in motor performance (*p* = 0.09, median differences = −0.67, 95% CI: −1.46–0.00), satisfaction (*p* = 0.85, median differences = 0.00, 95% CI: −1.00–1.00), motor quality (*p* = 0.11, median differences = 0.37, 95% CI: 0.00–1.00), and general motor skill ability (*p* = 0.76, median differences = 0.69, 95% CI: −3.00–4.00) after intervention. We conducted a pretest-posttest comparison for the whole cohort (*n* = 78) and results were in agreement with diagnosis-specific results (Supplementary Table 3).

### Posttest-only analysis

Comparing the post-test of the treatment group with the pre-test of the waitlist group in all three outcome measures confirm the result of pre-post analysis showing improved self-perceived motor performance and satisfaction as well as improved motor quality (*p < *0.001) but not general motor skill ability (Table 3). We combined children with developmental coordination disorder with and without attention deficit hyperactivity disorder in one group and ran this analysis comparing children in treatment and waitlist groups. Results are consistent with group specific results (Supplementary Table 4).

**Table 3.** Cognitive Orientation to daily Occupational Performance effect in treatment group compared to waitlist: posttest-only analysis.

Variable	DCD (n = 37)				DCD + ADHD (n = 41)			
	Treatment (*n* = 17) Median (IQR)	Waitlist (*n* = 20) Median (IQR)	Effect size	Median 95% CI	Treatment (*n* = 22) Median (IQR)	Waitlist (*n* = 19) Median (IQR)	Effect size	Median 95% CI
COPM Performance	7.0 (2.0)	3.3 (2.0)	0.56*	3.00–4.67	6.8 (2.0)	3.0 (2.0)	0.57*	3.33–5.00
COPM Satisfaction	8.0 (1.3)	3.3 (2.3)	0.59*	4.33–6.00	7.5 (2.9)	3.3 (2.3)	0.57*	3.16–5.33
PQRS	6.7 (2.2)	3.0 (1.7)	0.54*	2.33–4.67	5.4 (3.1)	3.3 (1.7)	0.46*	1.67–4.00
BOT-2 (percentile)	12 (16.5)	14 (16.0)	0.02	−6.00–6.00	17.5 (20.0)	16 (23.0)	0.01	−12.00–6.00

ADHD: attention deficit hyperactivity disorder; BOT-2: Bruininks-Oseretsky Test of Motor Proficiency – 2nd edition; COPM: Canadian Occupational Performance Measure; DCD: developmental coordination disorder; IQR: inter-quartile range; PQRS: Performance Quality Rating Scale.

* Bonferroni-corrected *p *< 0.001.

### Follow-up analysis

**Figure 3.**
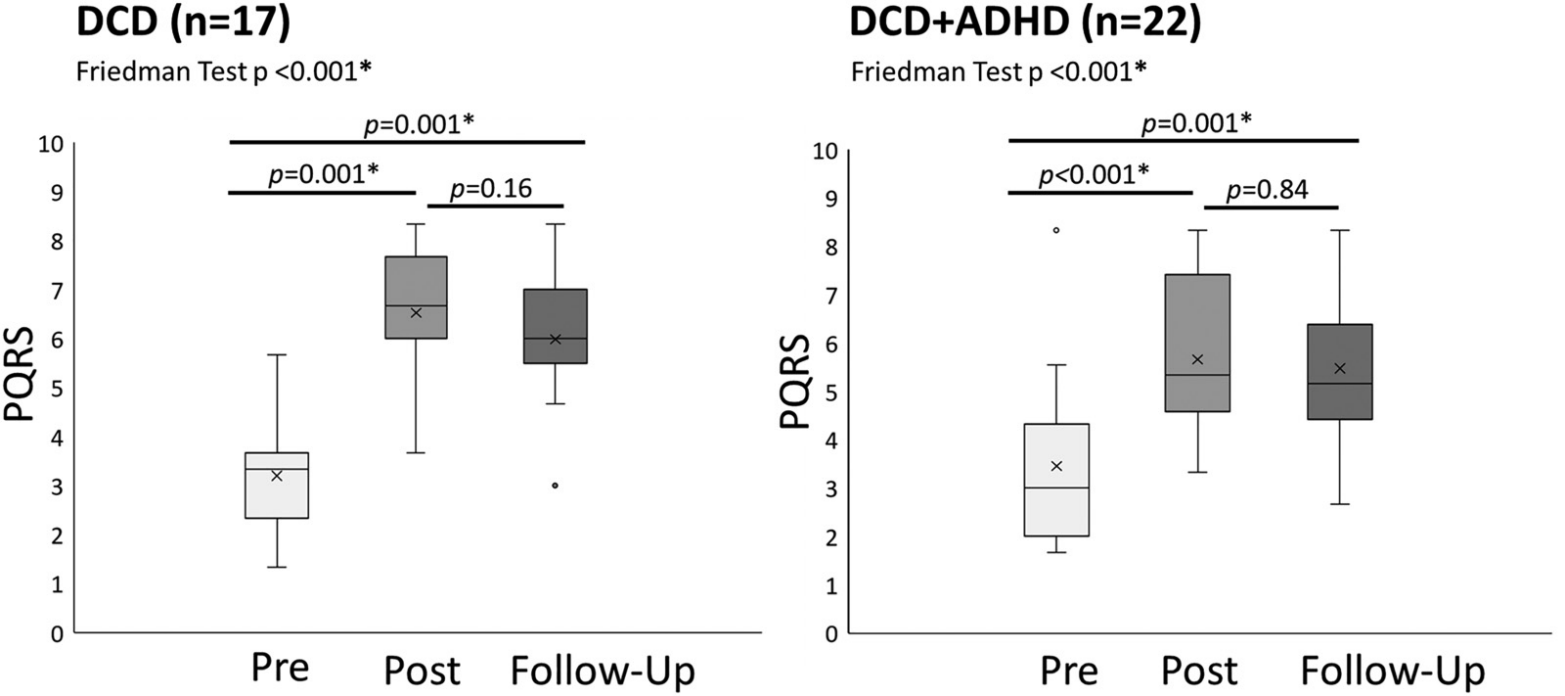
PQRS outcomes for treatment group. ADHD: attention deficit hyperactivity disorder; DCD: developmental coordination disorder; PQRS: Performance Quality Rating Scale. * significant after Bonferroni correction; x represents mean; line represents median.

**Figure 4.**
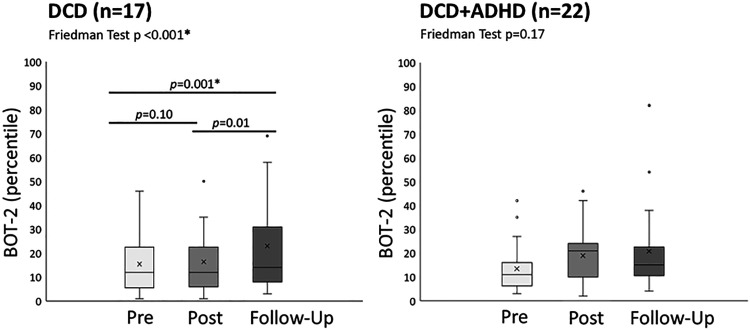
BOT-2 outcomes for treatment group. ADHD: attention deficit hyperactivity disorder; BOT-2: Bruninks-Oseretsky Test of Motor Proficiency – 2^nd^ ed.; DCD: developmental coordination disorder. * significant after Bonferroni correction; x represents mean; line represents median.

Analysis of follow-up data showed improved motor quality from pre-intervention to both post-intervention and follow-up assessments, while there was no significant difference between post-intervention and follow-up assessments (Table 4, Figure 3). For the Bruininks-Oseretsky Test of Motor Proficiency – 2^nd^ ed., there was a significant increase (*p < *0.001, effect size = 0.6) in general motor skill ability from pre-intervention to follow-up assessment in children with developmental coordination disorder only (Figure 4). Combined group results are reported in Supplementary Table 5. Spearman's correlation did not show a significant correlation between in-home practice (total minutes) and improvement in Bruininks-Oseretsky Test of Motor Proficiency – 2^nd^ ed. scores (*p* *=* 0.97 *r_s_* = 0.006) from pre-test to follow-up assessment.

**Table 4.** Motor outcomes of treatment group: follow-up analysis.

Variable	DCD (*n* = 17)				DCD + ADHD (*n* = 22)			
	Pre-test Median (IQR)	Post-test Median (IQR)	Follow-up Median (IQR)	Effect size	Pre-test Median (IQR)	Post-test Median (IQR)	Follow-up Median (IQR)	Effect size
PQRS	3.3 (1.3)	6.7 (1.7)	6.0 (1.2)	0.8*	3.0 (1.9)	5.5 (2.3)	5.2 (1.4)	0.4*
BOT-2 (percentile)	12 (15.0)	12 (12.0)	14 (20.0)	0.6*	11 (9.3)	21 (14.0)	15 (12.1)	0.08

ADHD: attention deficit hyperactivity disorder; BOT-2: Bruininks-Oseretsky Test of Motor Proficiency – 2nd edition; DCD: developmental coordination disorder; IQR: inter-quartile range; PQRS: Performance QualityRating Scale.

* Bonferroni-corrected *p *< 0.001.

## Discussion

Our findings support existing literature indicating the Cognitive Orientation to daily Occupational Performance intervention is effective in improving the self-perceived motor performance and satisfaction in child-chosen motor goals as well as quality of motor performance in children with developmental coordination disorder and children with developmental coordination disorder with co-occurring attention deficit hyperactivity disorder.^12,13,28,29^ As expected, the underlying motor skills assessed by the Bruininks-Oseretsky Test of Motor Proficiency – 2^nd^ ed. did not show a significant improvement immediately after the Cognitive Orientation to daily Occupational Performance intervention. However, in the follow-up assessment, children with developmental coordination disorder showed improvements in their quality of movement on the Performance Quality Rating Scale as well as their overall motor ability assessed by the Bruininks-Oseretsky Test of Motor Proficiency – 2^nd^ ed. when compared to their pre-intervention assessment. In contrast, children with developmental coordination disorder with co-occurring attention deficit hyperactivity disorder only showed improved goal-specific performance quality after three months. In what follows, we discussed our results in the context of main Cognitive Orientation to daily Occupational Performance objectives addressed in this study, including skill acquisition and transfer.^12^

To date, our study is the largest randomized controlled trial investigating the effectiveness of Cognitive Orientation to daily Occupational Performance intervention. In line with our results, several studies have reported the effect of cognitive task-based approaches such as Cognitive Orientation to daily Occupational Performance on “skill acquisition” of trained tasks in children with developmental coordination disorder,^12,13,29^ attention deficit hyperactivity disorder,^28^ autism spectrum disorder,^30^ and cerebral palsy.^31^ Moreover, children with developmental coordination disorder and those with co-occurring attention deficit hyperactivity disorder benefited from the intervention to a similar extent, as we did not observe any difference between the two groups. We reported that 90% to 92% of participants with developmental coordination disorder with co-occurring attention deficit hyperactivity disorder showed clinically significant improvement in their motor performance and satisfaction. Similarly, Gharebaghy and colleagues demonstrated that almost 100% of children with attention deficit hyperactivity disorder had clinically significant improvement in their motor performance and satisfaction from both their own perspective and their parents.^28^ Moreover, Green and colleagues found that children with co-morbidities (e.g. attention deficit hyperactivity disorder, learning difficulty) still benefit from the Cognitive Orientation to daily Occupational Performance intervention while they may continue to have difficulties.^32^

More importantly, both children with developmental coordination disorder and children with developmental coordination disorder with co-occurring attention deficit hyperactivity disorder maintained their task-specific motor improvements three months after the intervention, highlighting maintenance of these skills after intervention had stopped. In line with our results, parents of children with developmental coordination disorder have reported increased use of previously learned motor skills and cognitive strategies seven to 13 months after intervention,^13^ highlighting the lasting treatment effect of Cognitive Orientation to daily Occupational Performance.

In our study, the Bruininks-Oseretsky Test of Motor Proficiency – 2^nd^ ed., as a standardized motor assessment, was used to assess transferability of Cognitive Orientation to daily Occupational Performance to “foundational skills”. The Bruininks-Oseretsky Test of Motor Proficiency – 2^nd^ ed. assesses proficiency in general motor skill abilities in four areas: fine manual control, manual coordination, body coordination, and strength and agility.^21^ Children with developmental coordination disorder did not show immediate improvement in their Bruininks-Oseretsky Test of Motor Proficiency – 2^nd^ ed. scores after Cognitive Orientation to daily Occupational Performance intervention, but over time, they showed statistically significant improvement (*r* = 0.6) in their general motor skills from pre-intervention to follow-up. While at least eight studies have investigated Cognitive Orientation to daily Occupational Performance's transferability in children with developmental coordination disorder,^33^ the Bruininks-Oseretsky Test of Motor Proficiency – 2^nd^ ed. has been only used in two other studies;^13,34^ similar to our results, Chan (2007) did not find a significant difference in Bruininks-Oseretsky Test of Motor Proficiency – 2^nd^ ed. scores immediately after Cognitive Orientation to daily Occupational Performance while other measures of transferability (i.e. Assessment of Motor and Process Skills) showed different results.^34^ However, these results should be considered with caution considering their small sample size (*n* = 6) and that they did not correct for multiple comparisons.

Contrary to our results, Miller and colleagues showed immediate improvement in underlying motor difficulty after Cognitive Orientation to daily Occupational Performance.^13^ This contradiction can be explained by differences in statistical analysis and lack of correction for multiple comparisons. Integrating our results with the developmental coordination disorder literature might be an indication that Bruininks-Oseretsky Test of Motor Proficiency – 2^nd^ ed. is not sensitive enough to capture motor and foundational skills transfer immediately following Cognitive Orientation to daily Occupational Performance. Thornton and colleagues, on the other hand, used a more sensitive method to measure foundational skills (i.e. motor overflow measured by three-dimensional motion analysis).^35^ They determined that Cognitive Orientation to daily Occupational Performance not only improves activity and participation of children with developmental coordination disorder, but also reduces the underlying motor impairment immediately after intervention.^35^ Accordingly, although Cognitive Orientation to daily Occupational Performance primarily focuses on activity and participation, its role in improving body function, either immediate or delayed cannot be ignored.^35^ More studies are needed to confirm this hypothesis.

Unlike children with developmental coordination disorder, children with developmental coordination disorder with co-occurring attention deficit hyperactivity disorder did not show improvements in their general motor skills ability immediately following intervention or three months after intervention. In contrast, Gharebaghy and colleagues were the first to report improved Bruininks-Oseretsky Test of Motor Proficiency – 2^nd^ ed. scores immediately after Cognitive Orientation to daily Occupational Performance intervention in six children with attention deficit hyperactivity disorder.^28^ These discrepant findings may be because of the small sample size and the level IV of evidence in this single case experimental study design.^36^

Since there was no difference between children with developmental coordination disorder with or without co-occurring attention deficit hyperactivity disorder in their baseline motor ability and the frequency and type of selected goal during the intervention were similar in these two groups, we believe that motor problem severity and motor goals do not explain difference in transfer of motor learning. It is more likely that greater difficulty with attention and executive function in children with co-occurring attention deficit hyperactivity disorder may account for decreased ability to transfer motor learning to other tasks. Gharebaghy et al. suggest modifications to the Cognitive Orientation to daily Occupational Performance approach, such as providing rest time or free play between tasks, using playfulness strategies, role-playing, and repeating domain-specific strategies at the beginning of the session may be beneficial for children with attention deficit hyperactivity disorder.^28^ These adaptations may help increase attention and participation for children with developmental coordination disorder with co-occurring attention deficit hyperactivity disorder and subsequently affect its long-term effects and skill transference.

The results obtained in this study may be limited by several factors. A few participants were mistakenly randomized and included in our analysis which might have biased the results; we performed intention-to-treat analysis in order to resolve this issue. A limitation of our study is lack of behavioural comparator at baseline as another control for maturation in the waitlist group. Another limitations include the short follow-up, lack of Canadian Occupational Performance Measure at the follow-up assessment, and fewer participants in the follow-up analyses; to our knowledge, however, this is the largest cohort to data to report follow-up data. Using a standardized motor assessment (i.e. Bruininks-Oseretsky Test of Motor Proficiency – 2^nd^ ed.) to assess Cognitive Orientation to daily Occupational Performance transferability may not be the best assessment of transference. This could be improved in future studies by adding a fourth goal that is not addressed in therapy to see if the child can generalize the strategies that they learn to another, untrained motor goal. Furthermore, we did not have the opportunity to examine the generalizability of Cognitive Orientation to daily Occupational Performance to other environments. Lastly, we were not able to control for the potential effect of attention deficit hyperactivity disorder-related medications on our results due to the limitations of non-parametric statistics.

Despite these limitations, this study is the largest randomized controlled trial to date and provides high-level evidence to support Cognitive Orientation to daily Occupational Performance intervention for children with developmental coordination disorder, including those with co-occurring attention deficit hyperactivity disorder. Results showed that the intervention was effective in improving self-perceived motor performance and therapist-observed motor quality, and that these functional motor improvements were maintained several months after intervention. This study also provides some evidence that the Cognitive Orientation to daily Occupational Performance approach may help the transference of motor skills in children with developmental coordination disorder, but not in children with co-occurring attention deficit hyperactivity disorder. Given the executive function deficits associated with attention deficit hyperactivity disorder, children with a dual diagnosis of developmental coordination disorder and attention deficit hyperactivity disorder may need additional support and/or longer intervention to be able to transfer motor learning to other tasks. Future studies could investigate whether modifications to the Cognitive Orientation to daily Occupational Performance approach facilitate generalization and transfer in this group of children.

Findings from this study, together with accumulating evidence on the effectiveness of Cognitive Orientation to daily Occupational Performance intervention, may support advocacy efforts to provide this intervention as standard of care for children with developmental coordination disorder, with and without co-occurring attention deficit hyperactivity disorder.

**Clinical messages**

- The Cognitive Orientation to Occupational Performance is an effective intervention for children with developmental coordination disorder, with or without clinically significant attentional difficulties, with motor gains lasting for at least 3 months.
- This approach may transfer to foundational motor skills in children with developmental coordination disorder, but not in children with co-occurring attentional difficulties.

## Supplemental Material

sj-pdf-1-cre-10.1177_02692155221086188 - Supplemental material for Effectiveness of Cognitive Orientation to Occupational Performance intervention in improving motor skills of children with developmental coordination disorder: A randomized waitlist-control trial Supplemental material, sj-pdf-1-cre-10.1177_02692155221086188 for Effectiveness of Cognitive Orientation to Occupational Performance intervention in improving motor skills of children with developmental coordination disorder: A randomized waitlist-control trial by Sara Izadi-Najafabadi, Cassandra Gunton, Zara Dureno and Jill G Zwicker in Clinical Rehabilitation

## References

[1] Zwicker JG Missiuna C Harris SR , et al. Developmental coordination disorder: a review and update. Eur J Paediatr Neurol 2012; 16: 573–581. 22705270 10.1016/j.ejpn.2012.05.005

[2] American Psychiatric Association. Diagnostic and statistical manual of mental disorders. Washington, DC: American Psychiatric Publishing, 2013.

[3] Kirby A Sugden D Purcell C . Diagnosing developmental coordination disorders. Arch Dis Child 2014; 99: 292–296. 24255567 10.1136/archdischild-2012-303569

[4] Piek JP Pitcher TM Hay DA . Motor coordination and kinaesthesis in boys with attention deficit–hyperactivity disorder. Dev Med Child Neurol 1999; 41: 159–165. 10210248 10.1017/s0012162299000341

[5] Rasmussen P Gillberg C . Natural outcome of ADHD with developmental coordination disorder at age 22 years: a controlled, longitudinal, community-based study. J Am Acad Child Adolesc Psychiatry 2000; 39: 1424–1431. 11068898 10.1097/00004583-200011000-00017

[6] Missiuna C Cairney J Pollock N , et al. Psychological distress in children with developmental coordination disorder and attention-deficit hyperactivity disorder. Res Dev Disabil 2014; 35: 1198–1207. 24559609 10.1016/j.ridd.2014.01.007

[7] Flapper BC Schoemaker MM . Effects of methylphenidate on quality of life in children with both developmental coordination disorder and ADHD. Dev Med Child Neurol 2008; 50: 294–299. 18352997 10.1111/j.1469-8749.2008.02039.x

[8] Dewey D Volkovinskaia A . Health-related quality of life and peer relationships in adolescents with developmental coordination disorder and attention-deficit–hyperactivity disorder. Dev Med Child Neurol 2018; 60: 711–717. 29611868 10.1111/dmcn.13753

[9] Hoza B . Peer functioning in children with ADHD. J Pediatr Psychol 2007; 32: 655–663. 17556400 10.1093/jpepsy/jsm024

[10] Fliers EA Franke B Lambregts-Rommelse NN , et al. Undertreatment of motor problems in children with ADHD. Child Adolesc Ment Health 2010; 15: 85–90. 10.1111/j.1475-3588.2009.00538.x PMC2850122 20376200

[11] Fliers EA Franke B Buitelaar JK . Motor problems in children with ADHD receive too little attention in clinical practice. Ned Tijdschr Geneeskd 2011; 155: A3559–A3559. 22186361

[12] Polatajko HJ Mandich AD Miller LT , et al. Cognitive orientation to daily occupational performance (CO-OP): part II – the evidence. Phys Occup Ther Pediatr 2001; 20: 83–106. 11345514

[13] Miller LT Polatajko HJ Missiuna C , et al. A pilot trial of a cognitive treatment for children with developmental coordination disorder. Hum Mov Sci 2001; 20: 183–210. 11471396 10.1016/s0167-9457(01)00034-3

[14] Scammell EM Bates SV Houldin A , et al. The cognitive orientation to daily occupational performance (CO-OP): a scoping review. Can J Occup Ther 2016; 83: 216–225. 27301479 10.1177/0008417416651277

[15] Kleim JA Jones TA . Principles of experience-dependent neural plasticity: implications for rehabilitation after brain damage. 2008. 10.1044/1092-4388(2008/018) 18230848

[16] Izadi-Najafabadi S Gill KK Zwicker JG . Training-induced neuroplasticity in children with developmental coordination disorder. Curr Dev Disord Rep 2020; 7: 48–58.

[17] Izadi-Najafabadi S Rinat S Zwicker JG . Brain functional connectivity in children with developmental coordination disorder following rehabilitation intervention. Pediatr Res 2021: 1–10. 10.1038/s41390-021-01517-3 PMC9197764 33934120

[18] Blank R Barnett AL Cairney J , et al. International clinical practice recommendations on the definition, diagnosis, assessment, intervention, and psychosocial aspects of developmental coordination disorder. Dev Med Child Neurol 2019; 61: 242–285. 30671947 10.1111/dmcn.14132 PMC6850610

[19] Law M Baptiste S Carswell A , et al. Canadian Occupational performance measure (COPM). 5th ed. Toronto, ON: CAOT Publication ACE, 2014.

[20] Martini R Rios J Polatajko H , et al. The performance quality rating scale (PQRS): reliability, convergent validity, and internal responsiveness for two scoring systems. Disabil Rehabil 2015; 37: 231–238. 24766150 10.3109/09638288.2014.913702

[21] Bruininks R Bruininks B . Bruininks-Oseretsky test of motor proficiency. 2nd ed. Minneapolis, MN: NCS Pearson, 2005.

[22] Carswell A McColl MA Baptiste S , et al. The Canadian occupational performance measure: a research and clinical literature review. Can J Occup Ther 2004; 71: 210–222. 15586853 10.1177/000841740407100406

[23] Law M Baptiste S McColl M , et al. The Canadian occupational performance measure: an outcome measure for occupational therapy. Can J Occup Ther 1990; 57: 82–87. 10104738 10.1177/000841749005700207

[24] Mandich A Polatajko H Miller L , et al. The pediatric card sort. Ottawa, ON: CAOT Publications ACE, 2004.

[25] Polatajko HJ Mandich AD Missiuna C , et al. Cognitive orientation to daily occupational performance (CO-OP): part III – the protocol in brief. Phys Occup Ther Pediatr 2001; 20: 107–123. 11345506

[26] Mandich A Polatajko HJ . Enabling occupation in children: the cognitive orientation to daily occupational performance (CO-OP) approach. Ottawa, ON: CAOT Publications ACE, 2004.

[27] Capistran J Martini R . Exploring inter-task transfer following a CO-OP approach with four children with DCD: a single subject multiple baseline design. Hum Mov Sci 2016; 49: 277–290. 27522644 10.1016/j.humov.2016.07.004

[28] Gharebaghy S Rassafiani M Cameron D . Effect of cognitive intervention on children with ADHD. Phys Occup Ther Pediatr 2015; 35: 13–23. 25246134 10.3109/01942638.2014.957428

[29] Zwicker JG Rehal H Sodhi S , et al. Effectiveness of a summer camp intervention for children with developmental coordination disorder. Phys Occup Ther Pediatr 2015; 35: 163–177. 25229503 10.3109/01942638.2014.957431

[30] Rodger S Brandenburg J . Cognitive orientation to (daily) occupational performance (CO-OP) with children with asperger’s syndrome who have motor-based occupational performance goals. Aust Occup Ther J 2009; 56: 41–50. 20854488 10.1111/j.1440-1630.2008.00739.x

[31] Ghorbani N Rassafiani M Izadi-Najafabadi S , et al. Effectiveness of cognitive orientation to (daily) occupational performance (CO-OP) on children with cerebral palsy: a mixed design. Res Dev Disabil 2017; 71: 24–34. 28987969 10.1016/j.ridd.2017.09.007

[32] Green D Chambers M Sugden D . Does subtype of developmental coordination disorder count: is there a differential effect on outcome following intervention? Hum Mov Sci 2008; 27: 363–382. 18400322 10.1016/j.humov.2008.02.009

[33] Houldin A McEwen SE Howell MW , et al. The cognitive orientation to daily occupational performance approach and transfer: a scoping review. OTJR Occup Particip Health 2018; 38: 157–172. 10.1177/1539449217736059 29083265

[34] Chan DY . The application of cognitive orientation to daily occupational performance (CO-OP) in children with developmental coordination disorder (DCD) in Hong Kong: a pilot study. Hong Kong J Occup Ther 2007; 17: 39–44.

[35] Thornton A Licari M Reid S , et al. Cognitive orientation to (daily) occupational performance intervention leads to improvements in impairments, activity and participation in children with developmental coordination isorder. Disabil Rehabil 2016; 38: 979–986. 26213242 10.3109/09638288.2015.1070298

[36] Nielsen SK Kelsch K Miller K . Occupational therapy interventions for children with attention deficit hyperactivity disorder: a systematic review. Occup Ther Ment Health 2017; 33: 70–80.

