# Supplementary material for: Effectiveness of Cognitive Orientation to Occupational Performance intervention in improving motor skills of children with developmental coordination disorder: A randomized waitlist-control trial
Source: Clin Rehabil. 2022 Apr 24;36(6):776–88. doi: 10.1177/02692155221086188 (PMC9082975; doi:10.1177/02692155221086188)
Supplement: sj-pdf-1-cre-10.1177_02692155221086188 - Supplemental material for Effectiveness of Cognitive Orientation to Occupational Performance intervention in improving motor skills of children with developmental coordination disorder: A randomized waitlist-control trial [file sj-pdf-1-cre-10.1177_02692155221086188.pdf]

Effectiveness of Cognitive Orientation to Occupational Performance Intervention in Improving Motor Skills of Children with Developmental Coordination Disorder: A Randomized Waitlist-Control Trial

Supplemenary Material

Sample size calculation

A sample size calculation suggested that a sample of 25 participants per group (i.e., developmental coordination disorder and developmental coordination disorder with co-occurring attention deficit hyperactivity disorder) would have a power of 80% to detect clinically significant improvement of 2 points in self-perceived performance and satisfaction as assessed by our primary outcome measure, the Canadian Occupational Performance Measure,[1] with a standard deviation of 2.5 and a type-1 error of 0.05.

Participants

Developmental coordination disorder diagnosis was confirmed as per international guidelines[2] as follows: (1) a score of ≤16th percentile in the Movement Assessment Battery for Children – 2nd ed. (*Criterion A: motor skills below the expected level for age*);[3] (2) an age-dependent total score of 15 to 57 on the Developmental Coordination Disorder Questionnaire (*Criterion B: motor difficulties impact activities of daily living and academic performance*);[4] (3) parent report of motor difficulties from a young age (*Criterion C*); and (4) having no other medical condition based on medical examination, clinical judgement, and/or parent report (e.g., cerebral palsy, intellectual disability; *Criterion D*). Additional inclusion criteria were being between 8 to12 years of age and English-speaking. Attention deficit hyperactivity disorder

diagnosis was based on parent report. Children were excluded from the study if diagnosed with other neurodevelopmental disorders (e.g., autism spectrum disorder) or born pre-term (gestation week < 37 weeks). The Conner's Attention Deficit Hyperactivity Disorder Index – parent report form was used to quantify attention deficit hyperactivity disorder symptoms in this cohort.[5]

Procedure

Two registered occupational therapist not involved in the intervention conducted and scored pre- and post-intervention assessments – Canadian Occupational Performance Measure,[1] Performance Quality Rating Scale[6] and Bruininks-Oseretsky Test of Motor Proficiency 2nd edition[7] – for all participants. A 3-month follow-up assessment using the Performance Quality Rating Scale and Bruininks-Oseretsky Test of Motor Proficiency – 2nd ed. was conducted for children with developmental coordination disorder with or without attention deficit hyperactivity disorder in the treatment group. During the pre-intervention assessment session, children used the Pediatric Activity Card Sort (PACS)[8] to select three personal goals to be addressed over the course of treatment. The PACS consists of cards depicting childhood activities in various categories (e.g., personal care, school/productivity, hobbies/social activities). Some examples of goals the children chose included tying shoes, printing, and using a knife. Leisure-related goals, such as playing basketball, skipping a rope, and throwing and catching a ball were the most commonly chosen goals, followed by productivity and self-care goals respectively. Using chi-square analysis, we did not find a significant difference ($p > 0.05$) between frequency of goal types in developmental coordination disorder and developmental coordination disorder with co-occurring attention deficit hyperactivity disorder groups. Supplementary Table 1 shows a list of goal types and their frequencies in each group.

Supplementary Table 1. Type and Frequency of Selected Goals

	Goals	DCD (n=37)	DCD+ADHD (n=41)
**Leisure**	Badminton	3	2
	Basketball	13	12
	Throwing and catching	8	11
	Soccer/Football	8	6
	Skipping a rope	5	2
	Running	2	1
	Keyboarding	0	3
	Batting (hockey, baseball, golf)	1	4
	Frisbee	0	3
	Other	6	8
**Productivity**	Printing	15	16
	Handwriting and copying	9	12
	Typing	8	9
	Drawing	1	4
	Cutting with scissors	2	1
	Other	3	0
**Self-care**	Tying shoelaces	16	16
	Eating using cutlery	10	7
	Other	1	0

ADHD, attention deficit hyperactivity disorder; DCD, developmental coordination disorder.

Outcome measures

Using Canadian Occupational Performance Measure, children rated their motor performance and satisfaction for each of their three motor goals on a visual analog scale between 1 to 10, with higher scores indicating better performance and satisfaction; a two-point change is considered clinically significant.[9,10] In this study, assuming that Cognitive Orientation to daily Occupational Performance has a relatively similar effect on all goals and that all goals are to some extent related (all motor goals), an average of performance and satisfaction of all three goals pre- and post-intervention was calculated for data analysis purposes. The Canadian Occupational Performance Measure has a good face and content validity and is applicable for children over the age of 8 years.[10] It has good criterion responsiveness—ability to detect change over time—and moderate construct responsiveness—correlation with other measures.[11]

The Performance Quality Rating Scale objectively measures the quality of performance of child-selected and personally meaningful activities. Registered pediatric occupational therapists who administered the intervention videotaped children while performing their chosen goals immediately before their first intervention session and after their last intervention session. An occupational therapist assessor, blinded to the pre/post assessment sessions, watched the videos and rated the child's performance quality. The Performance Quality Rating Scale is composed of a 10-point performance rating scale where '1' indicates that the skill is not done at all and '10' indicates that the skill is performed very well. A change in score of 3 using the generic rating system is considered clinically significant.[6] The Performance Quality Rating Scale has been shown to be a precise tool with moderate to substantial inter-rater reliability, great test-retest reliability, and a good internal responsiveness, shown by large effect sizes for children with developmental coordination disorder.[6,12,13]

An independent occupational therapist, not involved in the intervention, used the short form of the Bruininks-Oseretsky Test of Motor Proficiency – 2nd ed. to objectively measure general motor skills on 14 items related to fine manual control, manual coordination, body coordination, and strength/agility. It has demonstrated moderate to strong inter-rater and test-retest reliability,[14] as well as excellent concurrent validity with other measures and adequate construct and content validity.[15] The total percentile score was used for analysis. An independent occupational therapist, not involved in the intervention, administered and scored the Bruininks-Oseretsky Test of Motor Proficiency – 2nd ed. to assessed the child's motor skill ability.

Statistical Analysis

We used the Shapiro-Wilk test to check normal distribution of continuous variables (age and Developmental Coordination Disorder Questionnaire) in each group. We reported mean and standard deviation (SD) for normally distributed variables, and median and interquartile range (IQR) for ordinal variables. A one-way ANOVA was used to compare normally distributed continuous variables (age and DCDQ) and nonparametric tests (i.e., Chi-square and Kruskal-Wallis H test) were used for ordinal variables (i.e., sex, scores on the Movement Assessment Battery for Children – 2nd ed. and Conner's Attention Deficit Hyperactivity Disorder Index, medications for Attention Deficit Hyperactivity Disorder) across groups. Since all outcome measures were ordinal – Canadian Occupational Performance Measure and Performance Quality Rating Scale are Likert scales and Bruininks-Oseretsky Test of Motor Proficiency – 2nd ed. percentile scores are ranked variables – non-parametric statistical tests were used to answer our research questions.

Continuous variables of age and Developmental Coordination Disorder Questionnaire were normally distributed in each group ($p > 0.05$), and thus are presented as means and SDs. All

other variables, including Movement Assessment Battery for Children – 2nd ed. percentile, Conner's Attention Deficit Hyperactivity Disorder Index, Canadian Occupational Performance Measure scores, Performance Quality Rating Scale scores, and Bruininks-Oseretsky Test of Motor Proficiency – 2nd ed. percentile rank, were ordinal; therefore, we reported median and inter-quartile range (IQR) for these variables.

To determine if the Cognitive Orientation to daily Occupational Performance intervention was effective in improving the self-rated measures of the Canadian Occupational Performance Measure and the objective measures of the Bruininks-Oseretsky Test of Motor Proficiency – 2nd ed. and Performance Quality Rating Scale, pre- and post-test scores for each participant with developmental coordination disorder and developmental coordination disorder with co-occurring attention deficit hyperactivity disorder were compared using Wilcoxon Signed-Rank Test. The Mann-Whitney U test was used to compare the changes (post-pre score) in all outcome measures between the two groups (developmental coordination disorder and developmental coordination disorder with co-occurring attention deficit hyperactivity disorder).

The posttest-only control group analysis was conducted to investigate the effect of maturation on motor performance of children with developmental coordination disorder with or without attention deficit hyperactivity disorder after Cognitive Orientation to daily Occupational Performance intervention. We compared the post-test scores of treatment group with the pretest of waitlist group in both developmental coordination disorder and developmental coordination disorder with co-occurring attention deficit hyperactivity disorder groups using Mann-Whitney U to confirm our results. Since group (treatment and waitlist) assignment was randomized and non-parametric tests are limited in controlling for covariates, we did not control for any of demographic variables when comparing treatment and waitlist groups.

To determine if the treatment was effective three-months post-intervention, the non-parametric Friedman Test was used to analyze data obtained from children with developmental coordination disorder and children with developmental coordination disorder with co-occurring attention deficit hyperactivity disorder in the treatment group (pre, post, and follow-up). R-value effect sizes were also calculated for both Wilcoxon Signed-Rank Test and Mann-Whitney U Test and the Kendall's W test value was calculated for Friedman tests.[16] For these analyses, alpha was set at 0.05 and, after correcting for multiple testing using Bonferroni, statistical significance was considered at p=0.001. To calculate Hodges-Lehman confidence intervals (CI), the confidence level was set at 95%. To deal with randomization errors and make an unbiased conclusion, we used an intention-to-treat approach.[17] Therefore, all randomized participants, regardless of their false or correct inclusion into the study and whether or not they completed the intervention and assessments, were included in the analysis according to their original group assignment. Since missing data pattern was random in our study, the "Data Imputation" method [18–20] was used to deal with the missing data of participants who did not complete the intervention or who did not attend the follow-up assessment. Using this approach, random numbers within the range of probable data were assigned to the missing data, and then data were analyzed. This process was repeated five times. The results were then pooled together to provide one final result.

Supplementary Table 2. Participant characteristics

Variable	DCD +/- ADHD (n=78)		*p-value*
	**Treatment (n=39)**	**Waitlist (n=39)**	
**Male Sex Assigned at Birth;** N (%)	32 (82)	31 (79)	0.8
**Age (years);** Mean (SD)	9.9 (1.4)	10.1 (1.5)	0.6
**DCDQ (total);** Mean (SD)	27 (10.5)	32 (11.5)	0.7
**MABC-2 (percentile);** Median (IQR)	1.0 (4.5)	2.0 (8.5)	0.7
**Conner's ADHD Index (t-score);** Median (IQR)	90 (4.5)	90 (4.0)	0.7
**ADHD-related Medications;** N (%)	11 (28)	15 (38)	0.3

ADHD, attention deficit hyperactivity disorder; DCD, developmental coordination disorder; DCDQ, Developmental Coordination Disorder Questionnaire; IQR, inter-quartile range; MABC-2, Movement Assessment Battery for Children – 2nd edition; SD, standard deviation.

Supplementary Table 3. Outcomes before and after Cognitive Orientation to daily Occupational Performance intervention: Pretest-Posttest analysis

Variable	DCD +/- ADHD (n=78)		*p-value*
	**Pre-test Median (IQR)**	**Post-test Median (IQR)**	
COPM Performance	2.7 (1.7)	7.0 (1.3)	< 0.00001
COPM Satisfaction	2.8 (2.3)	8.0 (2.0)	< 0.00001
PQRS	3.0 (1.7)	6.3 (2.3)	< 0.00001
BOT-2 (percentile)	12 (14.7)	16 (20.2)	0.002

ADHD, attention deficit hyperactivity disorder; BOT-2, Bruininks-Oseretsky Test of Motor Proficiency – 2nd edition; CI, confidence interval; COPM, Canadian Occupational Performance Measure; DCD, developmental coordination disorder; IQR, inter-quartile range; PQRS, Performance Quality Rating Scale.

Supplementary Table 4. Cognitive Orientation to daily Occupational Performance effect in treatment group compared to waitlist: Posttest-only analysis

Variable	DCD +/- ADHD (n=78)		*p-value*
	**Treatment (n=39) Median (IQR)**	**Waitlist (n=39) Median (IQR)**	
COPM Performance	7.0 (1.5)	3.0 (1.6)	< 0.00001
COPM Satisfaction	8.0 (1.8)	3.0 (2.3)	< 0.00001
PQRS	6.2 (2.3)	3.0 (1.2)	< 0.00001
BOT-2 (percentile)	12 (17.5)	14 (17)	0.9

ADHD, attention deficit hyperactivity disorder; BOT-2, Bruininks-Oseretsky Test of Motor Proficiency – 2nd edition; COPM, Canadian Occupational Performance Measure; DCD, developmental coordination disorder; IQR, inter-quartile range; PQRS, Performance Quality Rating Scale.

Supplementary Table 5. Motor outcomes of treatment group: Follow-up analysis

Variable	DCD +/- ADHD (n=39)			
	**Pre-test Median (IQR)**	**Post-test Median (IQR)**	**Follow-up Median (IQR)**	**Effect size**
PQRS	3.0 (1.8)	6.3 (3.0)	5.7 (2.1)	0.6*
BOT-2 (percentile)	10.0 (10.0)	14.0 (16.5)	14.0 (18.0)	0.3*

ADHD, attention deficit hyperactivity disorder; BOT-2, Bruininks-Oseretsky Test of Motor Proficiency – 2nd edition; DCD, developmental coordination disorder; IQR, inter-quartile range; PQRS, Performance Quality Rating Scale.

* Bonferroni-corrected p < 0.001

References

1. Law M, Baptiste S, Carswell A, McColl M, Polatajko H, Pollock N. Canadian Occupational Performance Measure (COPM). 5th ed. Toronto, ON: CAOT Publication ACE; 2014.

2. Blank R, Barnett AL, Cairney J, Green D, Kirby A, Polatajko H, et al. International clinical practice recommendations on the definition, diagnosis, assessment, intervention, and psychosocial aspects of developmental coordination disorder. Dev Med Child Neurol. 2019;61(3):242–85.

3. Henderson SE, Sugden DA, Barnett AL. Movement Assessment Battery for Children - 2nd ed.. Harcourt Assessment London; 2007.

4. Wilson BN, Kaplan BJ, Crawford SG, Roberts G. The Developmental Coordination Disorder Questionnaire 2007 (DCDQ'07). www.dcdq.ca

5. Conners CK. Conners 3. Toronto, ON: Multi-Health Systems; 2009.

6. Martini R, Rios J, Polatajko H, Wolf T, McEwen S. The Performance Quality Rating Scale (PQRS): reliability, convergent validity, and internal responsiveness for two scoring systems. Disabil Rehabil. 2015;37(3):231–8.

7. Bruininks R, Bruininks B. Bruininks-Oseretsky Test of Motor Proficiency. 2nd ed. Minneapolis, MN: NCS Pearson; 2005.

8. Mandich A, Polatajko H, Miller L, Baum C. The Pediatric Card Sort. Ottawa, ON: CAOT Publications ACE; 2004.

9. Carswell A, McColl MA, Baptiste S, Law M, Polatajko H, Pollock N. The Canadian Occupational Performance Measure: a research and clinical literature review. Can J Occup Ther. 2004;71(4):210–22.

10. Law M, Baptiste S, McColl M, Opzoomer A, Polatajko H, Pollock N. The Canadian Occupational Performance Measure: an outcome measure for occupational therapy. Can J Occup Ther. 1990;57(2):82–7.

11. Eyssen IC, Steultjens MP, Oud TA, Bolt EM, Maasdam A, Dekker J. Responsiveness of the Canadian Occupational Performance Measure. J Rehabil Res Dev. 2011;48(5):517–28.

12. Miller LT, Polatajko HJ, Missiuna C, Mandich AD, Macnab JJ. A pilot trial of a cognitive treatment for children with developmental coordination disorder. Hum Mov Sci. 2001;20(1–2):183–210.

13. Rodger S, Brandenburg J. Cognitive Orientation to (daily) Occupational Performance (CO-OP) with children with Asperger's syndrome who have motor-based occupational performance goals. Aust Occup Ther J. 2009;56(1):41–50.

14. Deitz JC, Kartin D, Kopp K. Review of the Bruininks-Oseretsky Test of Motor Proficiency, (BOT-2). Phys Occup Ther Pediatr. 2007;27(4):87–102.

15. Slater LM, Hillier SL, Civetta LR. The clinimetric properties of performance-based gross motor tests used for children with developmental coordination disorder: a systematic review. Pediatr Phys Ther. 2010;22(2):170–9.

16. Tomczak M, Tomczak E. The need to report effect size estimates revisited. An overview of some recommended measures of effect size. Trends Sport Sci. 2014;21(1).

17. Yelland LN, Sullivan TR, Voysey M, Lee KJ, Cook JA, Forbes AB. Applying the intention-to-treat principle in practice: Guidance on handling randomisation errors. Clin Trials. 2015;12(4):418–23.

18. Quintero M, LeBoulluec A. Missing data imputation for ordinal data. Int J Comput Appl. 2018;181(5):10–6.

19. Leite W, Beretvas SN. The performance of multiple imputation for Likert-type items with missing data. J Mod Appl Stat Methods. 2010;9(1):8.

20. Fox-Wasylyshyn SM, El-Masri MM. Handling missing data in self-report measures. Res Nurs Health. 2005;28(6):488–95.
